# Silane and acid etch cross contamination of dentin and composite reduced µ-tensile bond strength

**DOI:** 10.2340/biid.v11.41933

**Published:** 2024-10-01

**Authors:** Sigfus Thor Eliasson, Jon Einar Dahl

**Affiliations:** a Nordic Institute of Dental Materials, Oslo, Norway; b Faculty of Odontology, School of Health Sciences, University of Iceland, Reykjavik, Iceland

**Keywords:** Adhesive, repair of restorations, tensile testing

## Abstract

**Objectives:**

To investigate whether acid etch contamination of silane-treated composite influenced repair bond strength and whether silane contamination on dentin influenced composite bond strength to dentin.

**Materials and methods:**

Forty composite blocks stored in water for 4 weeks were divided into four groups. Specimens in groups 1–3 were coated with Bis-Silane and contaminated with acid etch + water spray (group 1) or water spray (group 2). Group 3 was not contaminated. Group 4 was untreated. The occlusal third of 60 third molars was cut off, ground flat, and divided into three groups. After etching, the surfaces in groups A and B were contaminated with Bis-silane. The contaminated surfaces in group A were re-etched.

Each composite repair group and composite-dentin group was divided into two subgroups receiving Adper Scotchbond 1 XT or Clearfil SE Bond 2 adhesives followed by a composite build up. After ageing for 3 months, specimens were sectioned into 1.1 mm × 1.1 mm rods for tensile testing and strength calculated at fracture. The fracture was examined using microscope.

**Results:**

Bis-Silane surface treatment increased the repair bond strength. Contamination with acid reduced the strength of the repair bond. Similar results were obtained for both adhesives. Tooth surface contamination with silane reduced the bond strength between dentin and composite. Additional acid etching or water spray on silane contaminated dentin did not influence the weakened bond strength. Most fractures were adhesive type.

**Conclusions:**

Silane contamination on etched dentin and acid etch contamination on silanized composite surfaces significantly reduced tensile bond strength.

## Introduction

Advances in dental adhesives have changed restorative dentistry from Black’s ‘extension for prevention’ to ‘minimum intervention’ or ‘minimally invasive’ operative dentistry, where the goal is to save as much tooth structure as possible [1, 2]. Part of this ideology is repairing instead of replacing faulty restorations [3–5]. Repairing and refurbishing defective resin composite restorations has, in the last two decades, been adopted into the curriculum of many dental schools [6, 7]. It has also been demonstrated that repairing composite restorations increases longevity [8], and even that longevity of repaired and replaced restorations is comparable [9–11]. World Dental Federation (FDI) has recently published a policy statement on repair of restorations [12]. FDI stated that advances in adhesive dentistry have made restoration repair an integral part of minimally invasive dentistry and that repairs aim to increase tooth survival.

Since the introduction of resin composite materials, investigators have explored methods to repair these restorations by adding new composite to the old [13]. New composite can be retained through undercuts and micromechanical bonding into irregularities in the prepared surface and theoretically also be retained by chemical bonding to the filler particles and the organic matrix. Numerous reports have been published on the repair strength of resin composites, where the effect of different surface treatments of the original substrate was evaluated [5, 13]. In these investigations, adhesive was used as a wetting agent. The authors of this paper have published several reports on repair strength of new composite to aged composite. The main conclusions from our investigations are that the strongest repair bond would be attained by roughening the composite substrate with a diamond, cleaning the surface by an acid etch procedure, and then applying a two-part bis-silane followed by a very thin layer of adhesive [4, 5, 14].

In most instances where composite restorations have failed, the repair requires bonding to not only old composite surface but also to tooth structure. Some unintentional contamination that influence bond strength have been investigated [15-20] but the effect of silane contamination on dentin and other cross contamination when repairing resin composite restorations has not obtained much attention in dental literature. Few papers could be found, where the effect of silane contamination on dentin was investigated. It has been reported that the bond strength of composite to dentin was not adversely affected when the dentin was contaminated with silane before adhesive application [21-23]. On the other hand, dentin contamination with silane and other repair conditioning measures after etching and after priming significantly decreased bond strength [22, 24].

The purpose of this study was to evaluate the effect of surface contamination on composite repair bond strength and the bond between dentin and composite, respectively. The tested null hypotheses were (1) contaminating silanized composite surfaces with acid etch does not reduce repair µ-tensile bond strength and (2) contaminating etched dentin surfaces with bis-silane does not reduce µ-tensile bond strength to composite.

## Materials and methods

The dental materials used in this study are listed in Table 1.

**Table 1:** Materials used in the investigation.

Product	Manufacturer	Lot no	Expiry date
Filtek™ Supreme XTE Universal Restorative shade A2B	3M ESPE Dental Products St. Paul, MN 55144-1000, USA	NA53220	2022-04-28
Adper™ Scotchbond 1XT Adhesive	3M ESPE Dental Products St. Paul, MN 55144-1000, USA	NA43407	2022-05-28
Clearfil™ SE Bond 2	Kuraray Europe Gmbh, 65795 Hattersheim am Main, Germany	000091	2022-04-30
Bis-Silane™ 2-part Porcelain primer	BISCO, Inc., Schaumburg, IL 60193, USA	Part A 1900002403 Part B 1900002404	2022-04-01
Imprint™4 Light	3M Deutschland Gmbh Dental Products, 41453 Neuss, Germany	7882765	2023-04-29

### Fabrication of test specimens for composite-composite repair bond strength measurements

**Figure 1:**
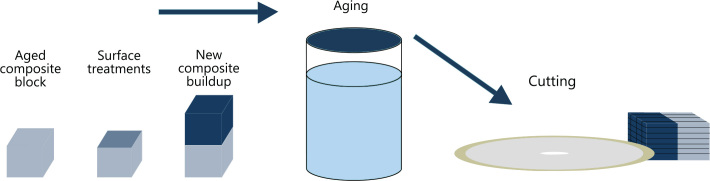
Schematic illustration showing preparation of test specimens for the composite-composite repair bond strength measurements.

The procedure and preparation of composite blocks are summarized in Figure 1. Forty Filtek Supreme XLT composite blocks, 10 mm × 6.2 mm wide and 8 mm high, were fabricated in customized Teflon molds in accordance with the manufacturer’s instructions. The composite blocks were incrementally built in four layers, and each layer cured for 40 s on each of three overlapping areas with a Demetron A2 corded LED curing light (Kerr Corp., Orange, CA. USA). The light output was measured to 1,100 mW/cm² (Norwegian Radiation Protection Authorities, Österaas, Norway).

After polymerization, the composite blocks were stored in distilled water for a total of 4 weeks [25, 26]. The specimen blocks were surfaced on a 320-grit silicon carbide sandpaper disc (Struers, Copenhagen, Denmark) under running water for 5 s to obtain a flat surface with standardized roughness like that of using a medium grid size diamond bur [4]. For cleaning purposes, the test surfaces were acid etched with 37% phosphoric gel for 15 s, rinsed with water for another 15 s, and gently air dried for 5 s.

The aged blocks were divided randomly into four experimental groups (Table 2). In groups 1–3, the test surfaces were coated with Bis-Silane, a two-part silane porcelain primer. The two parts were mixed and applied with a small brush for 30 s and gently dried with air for 5–10 s to evaporate the solvent. The silane coated surfaces were contaminated by acid etch and water spray (group 1) or water spray (group 2). In group 3, the silanized surfaces were not contaminated. In group 4, the surfaces were neither silanized nor contaminated.

**Table 2:** Experimental set-up for the composite-composite repair bond strength.

Base specimens	Filtek Supreme XLT shade A2 blocks							
Ageing	Water storage for 1 month							
Surface treatment 1	Sandpaper, 320 grid							
Surface treatment 2	Acid etch (37% phosphoric gel for 15 s) + water rinse (15 s)							
Group – Surface treatment 3	1 – Bis-Silane		2 – Bis-Silane		3 – Bis-Silane		4 – None	
Surface contamination	Acid etch + water rinse		Water rinse		None		None	
Surface treatment 4 (adhesive)	Adper Sotchbond 1XT	Clearfil SE Bond 2	Adper Scotchbond 1XT	Clearfil SE Bond 2	Adper Scotchbond 1XT	Clearfil SE Bond 2	Adper Scotch-bond 1XT	Clearfil SE Bond 2
Specimen designation	1a	1b	2a	2b	3a	3b	4a	4b
Repair composite	Filtek Supreme XLT shade A2							
Ageing	Water storage for 3 months							
Cutting	Preparing square test specimen rods approximately 1.1 X 1.1 mm.							
Number of test specimens	74	92	76	68	54	60	59	64

Each experimental group was divided into two subgroups that received either Filtek Scotchbond 1 XT, a one bottle total etch adhesive, or Clearfil SE Bond 2, a two-bottle self-etching adhesive (Table 2). The adhesives were applied and cured according to the manufacturer’s recommendations.

The original mold was placed over the aged surface treated composite blocks and the first layer of repair composite placed. To secure optimal adaptation of the repair material, the composite was placed in the middle of the specimen block to be repaired and adapted towards the margins with a small spatula. The second mold was then carefully fitted on the top and the composite further adapted and cured in total three approximately 2 mm incremental layers resulting in 14 mm high specimens. The repaired composite blocks were placed in distilled water for 3 months [25, 26].

### Fabrication of test specimens for dentin-composite bond strength measurements

**Figure 2:**
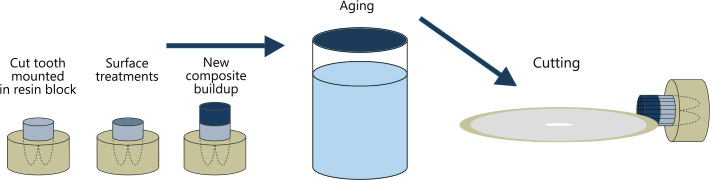
Schematic illustration showing preparation of test specimens for the composite-dentin bond strength measurements.

The preparation of dentin-composite specimens is summarized in Figure 2. Sixty sound third molars that had been surgically removed from around 20 years old individuals were obtained from a biobank at NIOM, with permission to be used for adhesive testing (Approved by Regional Committees for Medical and Health Research Ethics, Norway, #2014/457). The occlusal third of the crowns was cut off and ground flat on a 320-grit silicon carbide sandpaper disc (Struers, Copenhagen, Denmark) under running water to obtain a flat surface confined to superficial coronal dentin, as recommended by The Academy of Dental Materials and ISO/TS 11405 [27, 28]. The root portion of each tooth was mounted in a 25 mm in diameter and 15 mm high mold that was filled with self-curing resin.

The teeth were divided randomly into three groups A, B and C. The test surfaces of all the teeth were acid etched with 37% phosphoric gel for 15 s, rinsed with water for another 15 s and then blotted dry as recommended by the manufacturers of the adhesive used. In groups A and B, the etched surfaces were contaminated with Bis-Silane. The silane-contaminated surfaces in group A were re-etched and water sprayed.

Each experimental group (A, B, C) was then further divided into two subgroups, a and b, which received different adhesives, same as for the composite-composite repair (Table 3).

**Table 3:** Experimental set-up for the composite-dentin bond strength.

Base specimens	Tooth, ground into dentin, mounted in resin ring					
Surface treatment 1	Sandpaper, 320 grid					
Surface treatment 2	Acid etch (37% phosphoric gel for 15 s) + water rinse (15 s) + blot					
Group – Surface contamination	A – Bis-Silane		B – Bis-Silane		C – None	
Surface treatment 3	Acid etch + rinse with water		None		None	
Surface treatment 4 (adhesive)	Adper Scotchbond 1XT	Clearfil SE Bond 2	Adper Scotchbond 1XT	Clearfil SE Bond 2	Adper Scotchbond 1XT	Clearfil SE Bond 2
Specimen designation	Aa	Ab	Ba	Bb	Ca	Cb
Repair composite	Supreme XLT shade A2					
Ageing	Water storage for 3 months					
Cutting	Preparing square test specimen rods approximately 1.1 X 1.1 mm.					
Number of test specimens	129	155	160	151	138	141

Using customized Teflon mold, Filtek Supreme XTE resin composite was placed on the dentin surface in three 2 mm increments and cured, resulting in cylinder buttons, measuring 10 mm in diameter and 6 mm in height. After polymerization, the specimens were immediately stored in distilled water for 3 months [25, 26].

### Tensile testing

Both composite-composite blocks and tooth-composite specimens were mounted in an automatic cutting machine (Struers Secotom-60, Copenhagen, Denmark) equipped with a water-cooled thin diamond blade. The specimens were serially sectioned perpendicular to the bonding surface, both in the x- and the y-axis, producing square test specimen rods approximately 1.1 mm × 1.1 mm in thickness (Figures 1 and 2). After the first cut, light-bodied impression material (Permadyne Garant 2:1, 3MEspe Dental Products, MN, USA) was injected into the cuts for support before the second cut. Twelve to twenty-four specimen rods were obtained from each composite-composite and composite-tooth specimen. The test specimens were cleaned ultrasonically in distilled water for 3 min. After the cleaning procedure, the test specimen rods were examined light microscopically at a magnification of 40X for voids and imperfections (Nexius Zoom, Euromex, Netherlands). Only flawless specimen rods were used for the testing. The width and thickness of each test specimen were measured to the nearest 0.01 mm using a calibrated digital caliber (Mitutoyo Co, Kawasaki, Japan).

**Figure 3:**
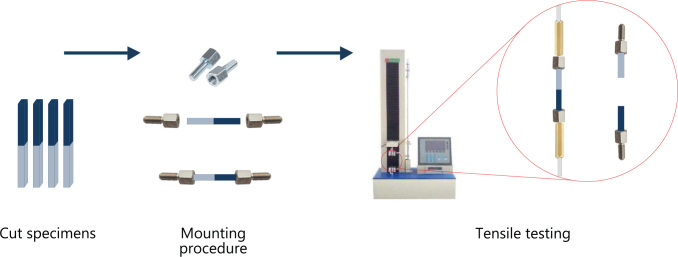
Schematic illustration of the mounting procedure and tensile testing of the test specimens. The female end of 2 mm standoff screws (ELRA AS, Oslo Norway) were fitted to each end of the 1.1 mm specimen rods and secured with cyanoacrylate glue (Locktide 435, Hankel Norden, Gothenburg, Sweden). A special fitting mold was made to insure alignment of the screws to the long axis of the specimen T. Each test specimen was mounted in a calibrated universal testing machine (Lloyd Instruments LTD, Model LRX, Fareham, England) using specially attached steel wires designed to transmit pure tensile forces to the specimen.

Our improved and less time consuming µ-tensile testing method, which has been described in previous scientific papers, was used for the tensile testing (Figure 3) [4, 5, 14]. Each test specimen was mounted in a calibrated universal testing machine (Lloyd Instruments LTD, Model LRX, Fareham, England) using specially attached steel wires designed to transmit pure tensile forces to the specimen. The testing was conducted at a crosshead speed of 1 mm/min until fracture. The tensile bond strength of each test specimen was calculated in MPa, by dividing the imposed force (in Newton) at fracture by the cross-sectional bond area (in mm²). All test specimens were maintained moist throughout the preparation and the test procedure.

The fracture surfaces were examined under a stereo microscope (Nexius Zoom, Euromex, Netherlands) at 40X magnification to determine whether the failure region was within the adhesive zone or out of it. The adhesive zone was defined as the interphase between the old and the new composite or between dentin and composite. Fracture in the adhesive zone was classified as adhesive failure and in the composite or dentin as cohesive failure.

**Figure 4:**
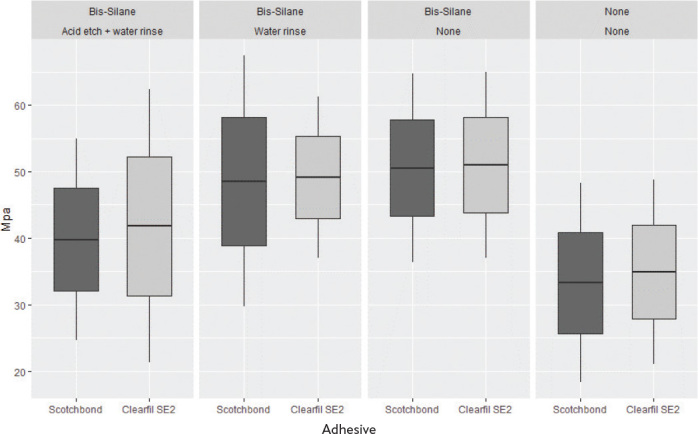
Box plot of composite repair strength measurements. The middle line is the mean. The lower part of the box is the mean minus standard deviation and the top part of the box is the mean plus standard deviation. The line depicts the 95 % confidence interval of the raw-data.

**Figure 5:**
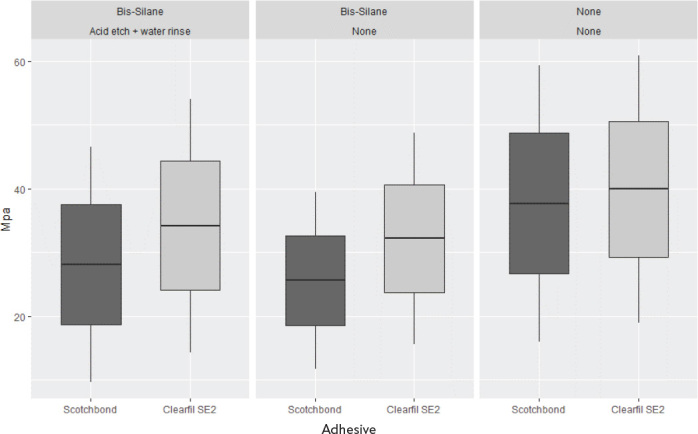
Box plot of composite-dentin bond repair strength measurements. The middle line is the mean. The lower part of the box is the mean minus standard deviation and the top part of the box is the mean plus standard deviation. The line depicts the 95 % confidence interval of the raw-data.

Statistical calculations were according to suggestions from ISO/TS 11405:2015 on treatment of results for testing of adhesion [27]. Each test specimen produced from the composite-composite blocs or dentin-composite specimen was regarded as statistical unit. The analysis was performed using the STATA SE version 16.1 (StataCorp LLC, College Station, TX) and R version 4.2.0 (R, Vienna, Austria). Shapiro-Wilk’s method was used to test for normality. Comparisons of mean bond strength were performed using Student’s *t*-test with significance level alpha <0.05. A Bonferroni correction was done on the *p*-values to account for multiple testing. Figures 4 and 5 were made using the ‘ggplot2’ package in R (v. 4.2.0).

## Results

### Composite-composite repair bond strength

The results are presented in Table 4 and Figure 4. There was no statistically significant difference in repair µ-tensile bond strength between the adhesives. The highest mean µ-tensile bond strength was in groups where there was no contamination of the silanized surface. Contaminating the silanized composite surfaces with water spray did not show statistically significant difference to not-contaminated surface. Contaminating the silanized composite surface with acid etching followed by rinsing with water spray significantly reduced the repair bond strength, but the strength was still significantly higher than that of the groups without silane treatment of the aged composite.

**Table 4:** Results of composite-composite repair bond strength evaluation.

Main Groups	1		2		3		4	
Surface treatment	Bis-Silane		Bis-Silane		Bis-Silane		None	
Surface contamination	Acid etch + water rinse		Water rinse		None		None	
Subgroups	1a	1b	2a	2b	3a	3b	4a	4b
Adhesive	Adper Scotchbond 1XT	Clearfil SE2	Filtec Scotchbond 1XT	Clearfil SE2	Filtec Scotchbond 1XT	Clearfil SE2	Filtec Scotchbond 1XT	Clearfil SE2
Number of specimen rods	74	92	76	68	54	60	59	64
Mean µTF (SD) in Mpa	39.8 (7.7)^Aa^	41.8 (10.5)^Ad^	48.5 (9.6)^Bb^	49.1 (6.2)^Be^	50.5 (7.2)^Cb^	51.0 (7.1)^Ce^	33.3 (7.6)^Dc^	34.9 (7.1)^Df^
Aggregate (a + b) mean µTF (SD) in Mpa	40.9 (9.4)^β^		48.8 (8.2)^α^		50.8 (7.1)^α^		34.1 (7.3)^δ^	
% µTF of strongest bond (3)	80.5		96.1		(100)		67.18	
% cohesive fracture in old composite	5.4	5.4	11.9	10.3	13.0	13.3	5.1	6.2
% cohesive fracture in new composite	1.4	1.1	2.6	2.9	1.9	1.7	0	0
% adhesive fracture	93.2	93.5	85.5	86.8	85.1	85.0	94.9	93.8

µTF (SD) in MPa: Micro Tensile Force (Standard Deviation) in Mega Pascals.

Different uppercase letters show statistical different values between adhesive subgroups within each main group.

Different lowercase letters show statistical different values between adhesive subgroups of main groups.

Different Greak letters show statistical different values between main groups (agregated values).

Most cohesive fractures in the old composite were in groups with the highest mean repair strength. The lowest portion of cohesive fractures in the old composite were in groups without silane-treated composite surface. Cohesive fracture in the new composite was rare (Table 4).

### Dentin-composite bond strength

The results are presented in Table 5 and Figure 5. The highest mean µ-tensile strength to dentin was in group where the etched dentin was not contaminated. Contamination of the surface with silane reduced the bond strength significantly and even re-etching the surface afterwards did not improve the strength significantly. Clearfil SE Bond 2 gave significantly higher µ-tensile strength values than Adper Scotchbond 1 XT when the dentin was contaminated. This difference between the adhesives was not observed when there was no contamination.

**Table 5:** Results of dentin-composite bond strength evaluation.

Main Groups	1		2		3	
Surface contamination	A – Bis-Silane		B – Bis-Silane		C – None	
Surface treatment	Acid etch + water rinse		None		None	
Subgroups	Aa	Ab	Ba	Bb	Ca	Cb
Adhesive	Adper Scotchbond 1XT	Clearfil SE Bond 2	Adper Scotchbond 1XT	Clearfil SE Bond 2	Adper Scotchbond 1XT	Clearfil SE Bond 2
Number of specimen rods	129	155	160	151	138	141
Mean µTF (SD) in MPa	28.1 (9.4)^Aa^	34.2 (10.1)^Bc^	25.6 (7.1)^Ca^	32.2 (8.5)^Dc^	37.7 (11.1)^Eb^	39.9 (10.7)^Ed^
Aggregated (a + b) mean µTF (SD) in MPa	31.4 (10.3)^α^		28.8 (8.4)^α^		38.8 (10.9)^β^	
% µTF of strongest bond (3)	80.9		74.2		(100)	
% cohesive fracture in dentin	3.1	3.2	1.9	0.7	1.4	2.8
% cohesive fracture in composite	7.8	6.5	5.6	4.6	11.6	12.1
% adhesive fracture	89.1	90.3	92.5	94.7	87.0	85.1

µTF (SD) in MPa: Micro Tensile Force (Standard Deviation) in Mega Pascals.

Different uppercase letters show statistical different values between adhesive subgroups within each main group.

Different lowercase letters show statistical different values between adhesive subgroups of main groups.

Different Greak letters show statistical different values between main groups (agregated values).

Cohesive fractures in dentin were rarely observed (Table 5). Cohesive fractures in the composite were highest in groups with no silane contamination where also the repair bond was strongest.

## Discussion

This study evaluated the effect on bond strength of contamination of dentin and silanized composite when repairing composite restorations. When silanizing composite in the clinic, it is possible that surrounding dentin becomes inadvertently contaminated and that silanized composite becomes contaminated when tooth structure is etched and rinsed with water. The results showed that both null hypotheses were rejected.

In this investigation, silanizing the aged composite significantly improved repair bond strength. When the substrate composite was not silanized, the repair µ-tensile bond strength was only 67% of the bond strength obtained when the composite was silanized before repair. This is consistent with results from previous studies [4, 5].

Two quite different products, Adper Scotchbond 1 XT, an established one bottle total-etch adhesive and Clearfil SE Bond 2, a two-step primer and bond self-etch system, were used in this investigation. In all sub-groups, Clearfil SE Bond 2 gave somewhat higher µ-tensile bond strength than Adper Scotchbond 1 XT even though the difference was not statistically significant except when the dentin was contaminated with Bis-silane. The thickness of the Clearfil SE Bond 2 bonding layer was observed to be only half of the thickness obtained with Adper Scotchbond 1 XT and might have contributed to the slightly higher bond strength. In previous studies on repair bond strength, we found that the thinner the bonding layer, the stronger the repair bond [4, 5].

The µ-tensile strength was significantly reduced when the silane treated composite was contaminated by the acid etch procedure. The etching possibly altered or removed some of the silane and, therefore, lowered the bond strength. However, water spray followed by airdrying did not significantly reduce the repair bond strength. The composite surface was probably protected from effect of water spray due to the hydrophobic properties of the Bis-Silane.

The results showed that silane contamination on etched dentin significantly reduced µ-tensile bond strength. It was interesting that silane contamination affected the tensile bond strength using Adper Scotchbond 1 XT much more than that of Clearfil SE Bond 2. It was possible that the acidic primer for Clearfil SE Bond 2 penetrated the silane better and thereby increased the wettability of the contaminated dentin, leading to the higher bond strength values.

The effect of silane contamination is in accordance with the results of Soontornvatin et al. [22] who used tensile testing, while Chen et al. [21] measuring µ-shear strength, did not find significant reduction in composite bond strength to silane contaminated dentin. In the investigation of Chen et al. [21], most fractures were cohesive in dentin or composite that hided the factual adhesive strength. It was surprising that re-etching the silane contaminated dentin did not increase the adhesive strength to the level of not-contaminated dentin. Again, this could be due to the hydrophobic properties of the silane.

Investigating factual bond strength requires that the preparation of the test specimens and the selected test method result in fracture in the adhesive layer and not in the substates, i.e. composite or dentin. If a substantial portion of specimens fracture cohesively as has been seen after shear bond testing [29], little or no conclusion can be drawn from the results on repair strength. This is a problem with several research publications where 50% – 90% of specimens are reported to fracture cohesively [30–36]. Conclusions from such data that silane did not improve repair bond strength can therefore be misleading and based on misinterpretation of the data. The high percentile of adhesive fracture obtained in the present study increased the confidence of the results. Another strength of the present study was the large sample size. This success is attributed to our improved and much less time consuming µ-tensile test method described earlier [4, 5], where the attachment to the testing machine secured more straight alignment of the specimen rods, resulting in more uniform distribution of the tensile forces throughout the specimen.

Many authors have criticized shear bond tests for producing stress concentrations in the substrate leading to cohesive failure and recommended that µ-tensile testing should be used as laboratory research method [37–41]. This was supported in a review stating that µ-tensile test appeared to have a larger discriminative power than the traditional macro-shear test for clinical relevance [42]. On the other hand, shear testing is simple and easy to perform and still the most widely used method for testing adhesive strength [43, 44].

Based on this study, the following clinical recommendations may be suggested for restoration repairs involving composite and tooth structure: First, the remaining composite restoration should be silane coated. If the tooth structure is accidentally contaminated with silane, the contamination is removed using diamond bur under water spray. Next and depending on the adhesive to be used, the tooth part is either selective etched or total etched. Care should be taken to avoid etching gel on the silanized composite surface. After rinsing with water spray, the silanized surface is dried with air stream and the tooth part blotted or dried according to recommendations for the adhesive. After adhesive application, the restoration is repaired with composite.

## Conclusions

Contamination of composite and dentin surfaces prior to restoration repair reduced the repair strength.
